# Evaluation of mortality attributable to SARS-CoV-2 vaccine administration using national level data from Qatar

**DOI:** 10.1038/s41467-022-35653-z

**Published:** 2023-01-03

**Authors:** Adeel A. Butt, Mylai D. Guerrero, Elenor B. Canlas, Husni Al-Dwairi, Abeir Bakhiet Mohammed Ali Alimam, Abdur Rehman Mohamad, Mohammed Thamer Ali, Nidal Ahmad Asaad, Ali Ahmed Sheikh Saleh Alkeldi, Mohammad Fawaz Saber Mohammad, Anil G. Thomas, Abdullatif Al-Khal, Muna Al-Maslamani, Abdul-Badi Abou-Samra

**Affiliations:** 1 Corporate Quality and Patient Safety Department, Hamad Medical Corporation, Doha, Qatar; 2 Department of Medicine, Weill Cornell Medicine, New York, NY USA; 3 Department of Population Health Sciences, Weill Cornell Medicine, New York, NY USA; 4 Department of Medicine, Weill Cornell Medicine, Doha, Qatar; 5 Department of Population Health Sciences, Weill Cornell Medicine, Doha, Qatar; 6 Communicable Diseases Center, Hamad Medical Corporation, Doha, Qatar; 7 Heart Hospital, Hamad Medical Corporation, Doha, Qatar

**Keywords:** Outcomes research, Infectious diseases, Epidemiology, SARS-CoV-2

## Abstract

Accurate determination of mortality attributable to SARS-CoV-2 vaccination is critical in allaying concerns about their safety. We reviewed every death in Qatar that occurred within 30 days of any SARS-CoV-2 vaccine administration between January 1, 2021 and June 12, 2022. Probability of association with SARS-CoV-2 vaccination was determined by four independent trained reviewers using a modified WHO algorithm. Among 6,928,359 doses administered, 138 deaths occurred within 30 days of vaccination; eight had a high probability (1.15/1,000,000 doses), 15 had intermediate probability (2.38/1,000,000 doses), and 112 had low probability or no association with vaccination. The death rate among those with high probability of relationship to SARS-CoV-2 vaccination was 0.34/100,000 unique vaccine recipients, while death rate among those with either high or intermediate probability of relationship to SARS-CoV-2 vaccination was 0.98/100,000 unique vaccine recipients. In conclusion, deaths attributable to SARS-CoV-2 vaccination are extremely rare and lower than the overall crude mortality rate in Qatar.

## Abstract (web summary)

This study assesses the probability that deaths following a COVID-19 vaccination are attributable to the vaccine. The authors review all deaths that occurred within 30 days of vaccine receipt in Qatar until June 2022 and find that deaths attributable to vaccination are extremely rare.

## Introduction

Despite their demonstrated safety and efficacy^1–6^, COVID-19 vaccine hesitancy and refusal are not uncommon^7,8^. Though there are scant published data, deaths related to COVID-19 vaccination appear to be extremely rare. The Centers for Disease Control and Prevention (CDC) in the United States received 14,980 reports of death among recipients of >589 million vaccine doses between December 14, 2020 and June 6, 2022^9^. However, after a review of all available information, CDC identified only nine deaths to be causally associated with the J&J/Janssen COVID-19 vaccination^9^. A systematic review of published studies reporting autopsy data identified only 38 cases causally linked to COVID-19 vaccination, with 22 cases linked to ChAdOx1 nCoV-19, 10 cases to BNT162b2, 4 cases to mRNA-1273, and 2 cases to Ad26.COV2.S vaccines^10^. Lack of clear and universally accepted criteria are a major limitation in assessing COVID-19 vaccine related deaths, with the onus of assigning causality falling upon the reviewing physician. This likely leads to significant variations in determining the association between vaccination and deaths. We developed a modified algorithm using criteria suggested by the World Health Organization to investigate the relationship between mRNA COVID-19 vaccination and deaths in Qatar at a national level.

## Results

**Fig. 1:**
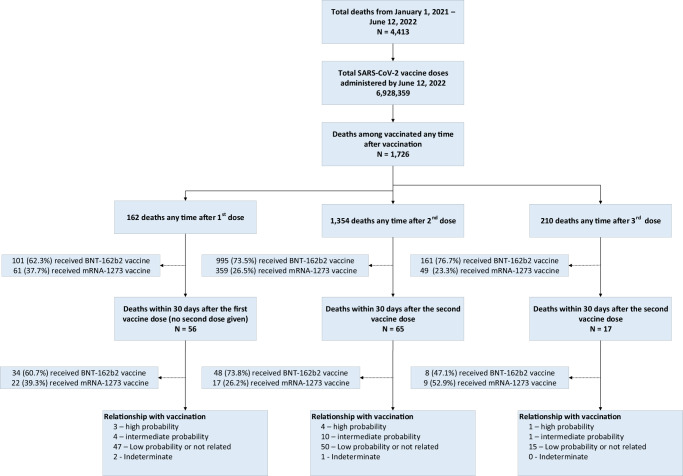
Algorithm used to classify the probability of death being associated with COVID-19 vaccination.

As of June 12, 2022, a total of 6,928,359 SARS-CoV-2 vaccine doses had been administered in Qatar^11^. Over 99% of the vaccines administered were the mRNA vaccines (BNT-162b2 and mRNA-1273). A total of 4413 deaths were recorded during the study period, with 1726 deaths occurring any time after at least one dose of a SARS-CoV-2 vaccine (162 deaths after 1 dose, 1354 deaths after 2 doses, and 210 deaths after 3 doses) (Fig. 1). Fifty-six deaths occurred within 30 days of receiving a first dose (with no second dose administered); 65 within 30 days of the second dose of the vaccine; 17 deaths occurred within 30 days of receiving the third dose of the vaccine (Fig. 1).

The median age of final study group (*N* = 138) was 55 years (IQR 43,70) and the median time from last vaccine to death was 17 days (IQR 11,22). Among them, 113 (81.9%) were male and 31 (22.5%) were Qatari nationals. Among the 138 deaths evaluated, 8 deaths were classified as having high probability of being related to vaccination (1.15 death/1,000,000 vaccine doses administered). Fifteen deaths were classified as having intermediate probability (2.16 death/1,000,000 vaccine doses administered). One hundred and twelve deaths were classified as low probability or not related to vaccination.

### Additional analyses

All cases determined to have a high, intermediate, or low probability of association with SARS-CoV-2 vaccination were further individually and separately reviewed by two trained mortality reviewers to assign the most proximal underlying cause(s) that precipitated the terminal event (not “mode of death”, i.e., cardiorespiratory arrest, sepsis without further classification, etc.) according to the World Health Organization criteria for assigning cause of death. Cause of death in all eight cases determined to have high probability of association with vaccination was “sudden cardiac death” in persons with no known risk factors for cardiac disease. Details of the underlying causes and contributory factors are provided in Supplementary Table 1.

Since time of death from the time of last vaccination is an arbitrary measure of association, we re-analyzed probability of association using a different time cut-off. Those with no risk factor for mortality were assigned high probability if the death occurred within 15 days, and intermediate probability if death occurred between 16–30 days of vaccine administration. Twenty-nine deaths were thus classified as high probability while 19 deaths were classified as intermediate probability of being related to vaccination.

## Discussion

To our knowledge, this is the first study to report the association between SARS-CoV-2 vaccination and death at a national level. Our results confirm the rarity of association between SARS-CoV-2 vaccination and death.

Crude death rates in Qatar for the years 2019, 2020 and 2021 were 6.60, 7.94, and 8.74 per 100,000 population^12^. Death rate among the vaccinated persons with high probability of relationship to SARS-CoV-2 vaccination was 0.34 per 100,000 vaccine recipients, while death rate among the vaccinated persons with either high or intermediate probability of relationship to SARS-CoV-2 vaccination was 0.98 per 100,000 (8 deaths classified as high probability and 15 deaths as intermediate probability among 2,347,635 unique persons who received at least one dose of a vaccine). While these are not age- or sex standardized mortality rates, they offer strong reassurance of extremely low rates of mortality attributable to SARS-CoV-2 vaccination, which is far below the overall crude mortality in the general population. With >90% of Qatar’s population having received at least two doses of the vaccine, the demographics of the vaccinated population are very likely to mirror those of the general population, excluding very young children who were not eligible for vaccination during the study period. There were 435 COVID-19 related deaths during the study period, with 90% occurring among the unvaccinated individuals, suggesting that the benefits of vaccination far outweigh the potential risks. For comparison, an age- and sex-specific breakdown of deaths in Qatar between 2019–2022 is provided in Supplementary Table 2.

The strengths of our study include availability of complete national data, a comprehensive review of each death, and use of trained medical personnel who used uniform data instruments to assign the probability of association between SARS-CoV-2 vaccination and death. Each case was reviewed by multiple reviewers to minimize individual bias. The major limitation is the lack of autopsies to determine the exact cause of death. Additionally, the time cut-offs chosen to determine the possible relationship were based on clinical experience and expert opinion of the authors since there are no evidence-based guidance available in this regard.

In conclusion, deaths attributable to SARS-CoV-2 mRNA vaccination are rare and the risk is lower than the overall crude mortality rate in Qatar. These results provide strong assurance regarding the safety of the mRNA vaccines. Our study also provides a strong framework and methodology for future studies to determine associations between vaccination and vaccine related deaths.

## Methods

### Study setting and study population

The study was conducted in the State of Qatar between January 1, 2021 and June 12, 2022. Qatar has a robust, centralized national mortality tracking system to examine all deaths in the country. All deaths in Qatar are individually reviewed by trained licensed physicians dedicated to mortality reviews who extract medical data from the electronic health records and assign direct, antecedent, and contributing causes of death for each case using the World Health Organization recommended definitions^13^. All deaths are processed in the public sector health facilities in Qatar which use an interconnected single electronic health records platform.

For the current study, medical records of all decedents during the study period who received any SARS-CoV-2 vaccine were reviewed. COVID-19 vaccination data for all decedents were retrieved from the Qatar National COVID-19 Database^3–6^. Those who had died within 30 days of receiving a SARS-CoV-2 vaccine dose were eligible for inclusion in the study.

### Vaccination and COVID-19 testing data

This study was conducted in the resident population of Qatar. COVID-19 laboratory testing, vaccination, and clinical infection data were extracted from the integrated, nationwide, digital-health information platform that hosts the national, federated severe acute respiratory syndrome coronavirus 2 (SARS-CoV-2) databases. These databases are complete with no missing information for polymerase chain reaction testing, COVID-19 vaccinations, COVID-19 hospitalizations, and basic demographic details, and have captured all SARS-CoV-2-related data since epidemic onset. Nearly all individuals were vaccinated in Qatar, through the universal public healthcare system for all nationals and residents of Qatar. For rare individuals who received COVID-19 vaccination outside Qatar, vaccination details were recorded in the health system at the port of entry upon return to Qatar, in order to fulfill national requirements and to benefit from privileges associated with vaccination, such as quarantine exemption^5,14–18^.

### Ascertainment of probability of death related to vaccination

**Fig. 2:**
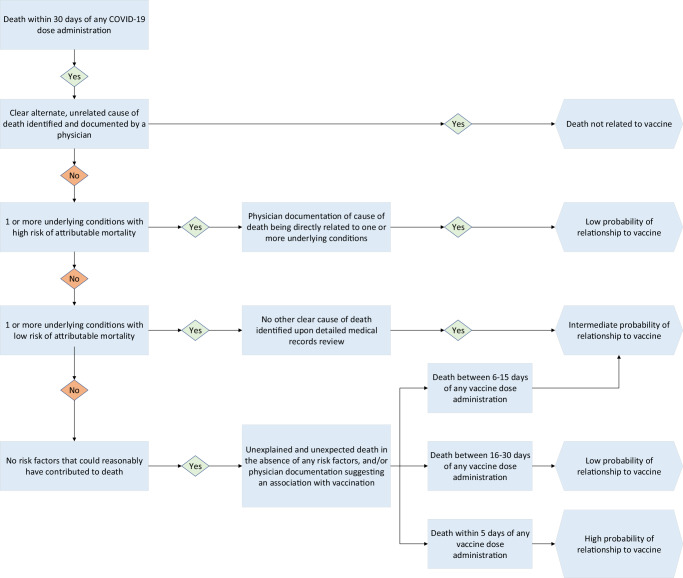
Study flow sheet demonstrating the individuals included in the analysis and the numbers of deaths with the probability of their relationship to SARS-CoV-2 vaccination.

We developed an algorithm to determine the probability of a death being related to COVID-19 vaccine administration using a framework similar to the one proposed by the World Health Organization, which takes into account evidence of any other cause of death, temporal association with vaccination, and lack of any other possible explanation^19,20^ (Fig. 2). Four licensed physicians were trained on the algorithm criteria to independently assign the probability of a death being related to vaccine administration. Final categorization was based on at least three reviewers assigning the same level of probability. Where less than three reviewers assigned the same probability of association, one additional reviewer adjudicated the outcome.

We created four categories of probability of association with SARS-CoV-2 vaccination: Not Related; Low Probability; Intermediate Probability; and High Probability. Cases with more than 2 levels of probability assigned by the four primary reviewers were classified as indeterminate. If a clear alternate and completely unrelated cause of death was identified (e.g., road traffic accident, suicide/homicide, widely metastatic cancer under palliative care), the death was classified as being not related to the vaccination. Presence of one or more severe underlying conditions associated with high risk of mortality (e.g., chronic advanced heart failure, pre-existing atherosclerotic heart disease with prior major adverse cardiovascular events) and physician documentation in the medical records of those contributing directly to death were used to assign low probability, while presence of one or more stable underlying conditions with low risk of short-term mortality and study reviewers’ confirmation of no clear alternate cause of death were used to assign intermediate probability of relationship to vaccination. Those with no underlying conditions and no plausible risk factor for death noted in the medical records or identified by reviewers were further subclassified based on time of death from last SARS-CoV-2 vaccination. Deaths occurring within 5 days of vaccination were classified as having high probability, deaths between 6–15 days as intermediate probability, and deaths occurring 16–30 days as having low probability of being causally associated with vaccination (Fig. 2).

All cases determined to have high or intermediate probability of association with SARS-CoV-2 vaccination were further reviewed to ascertain the underlying and contributory cause(s) of death by two trained mortality reviewers using the World Health Organization classification system. This review was specifically intended to identify the cause(s) that directly led to the precipitation of the terminal event that resulted in death.

Data were collected and analyzed in Microsoft Excel for Microsoft 365, Microsoft Corporation, Redmond, WA, USA.

### Ethical considerations

The study was approved by the Institutional Review Board at Hamad Medical Corporation. A waiver of informed consent was granted for the study since only decedents records were reviewed and all identifiers were subsequently removed.

### Reporting summary

Further information on research design is available in the Nature Portfolio Reporting Summary linked to this article.

## Supplementary information

Supplementary Information

Reporting Summary

## Data Availability

The national dataset used for this study is the property of the Qatar Ministry of Public Health that was provided to the researchers through a restricted-access agreement that prevents sharing the dataset with a third party or publicly. Future access to this dataset can be considered through a direct application for data access to Her Excellency the Minister of Public Health (https://www.moph.gov.qa/english/Pages/default.aspx). A.A.B. and A.A. had complete access to the data at all times and accept responsibility for the integrity of this article.
