# Supplementary material for: Evaluation of mortality attributable to SARS-CoV-2 vaccine administration using national level data from Qatar
Source: Nat Commun. 2023 Jan 3;14:24. doi: 10.1038/s41467-022-35653-z (PMC9808756; doi:10.1038/s41467-022-35653-z)
Supplement: Supplementary file 1 — Supplementary Information [file 41467_2022_35653_MOESM1_ESM.pdf]

Supplementary information

Article title: Probability of Deaths Related to the Administration of SARS-CoV-2 Vaccines: Evaluation of National Level Data from Qatar

Authors: Adeel A. Butt, Mylai D. Guerrero, Elenor B. Canlas, Husni Al-Dwairi, Abeir Bakhiet Mohammed Ali Alimam, Abdur Rehman Mohamad, Mohammed Thamer Ali, Nidal Ahmad Asaad, Ali Ahmed Sheikh Saleh Alkeldi, Mohammad Fawaz Saber Mohammad, Anil G. Thomas, Abdullatif Al-Khal, Muna Al-Maslamani, Abdul-Badi Abou-Samra

Contents:

Supplementary table 1. Underlying causes of death and contributory factors in persons determined have high, intermediate, or low probability of death being associated with SARS-CoV-2 vaccination. Age is presented in deciles of life to preserve anonymity due to small number of cases.

Supplementary table 2. Number of deaths in Qatar by age and sex between 2019-2021.

Supplementary table 1. Underlying causes of death and contributory factors in persons determined have high, intermediate, or low probability of death being associated with SARS-CoV-2 vaccination. Age is presented in deciles of life to preserve anonymity due to small number of cases.

Ser. No.	Probability of association	Age decile	No. of days between vaccination and death	Underlying cause of death	Contributory factors
**1.**	High Probability	41-50	3	Sudden cardiac death	No known comorbidities that may have contributed to death.
**2.**	High Probability	11-20	4	Sudden cardiac death	No known comorbidities that may have contributed to death.
**3.**	High Probability	41-50	3	Sudden cardiac death due to ventricular fibrillation.	No known comorbidities that may have contributed to death. Physician notes indicate the possibility of vaccine induced arrythmia.
**4.**	High Probability	51-60	1	Sudden cardiac death	No known comorbidities that may have contributed to death.
**5.**	High Probability	41-50	5	Sudden cardiac death	No known comorbidities that may have contributed to death. Suffered out-of-hospital cardiac arrest with no preceding symptoms recorded.
**6.**	High Probability	31-40	1	Sudden cardiac death	No known comorbidities that may have contributed to death. Suffered out-of-hospital cardiac arrest with no preceding symptoms recorded.
**7.**	High Probability	31-40	2	Sudden cardiac death	No known comorbidities that may have contributed to death. Had chest and epigastric pain 3 days prior to death but no cardiac diagnosis was recorded.
**8.**	High Probability	31-40	3	Sudden cardiac death	No known comorbidities that may have contributed to death.
**9.**	Intermediate Probability	61-70	10	Sudden cardiac death likely due to atherosclerotic heart disease	History of hypertension, diabetes mellitus, dyslipidemia, atherosclerotic heart disease, which were stable. Patient was found unresponsive at home.
**10.**	Intermediate Probability	41-50	14	Sudden cardiac death due to ventricular fibrillation	History of hypertension, diabetes mellitus, depression, and anxiety. Suffered out-of-hospital cardiac arrest and emergency responders noted ventricular fibrillation.
**11.**	Intermediate Probability	31-40	8	Sudden cardiac death	No known comorbidities that may have contributed to death. Suffered out-of-hospital cardiac arrest with no preceding symptoms.
**12.**	Intermediate Probability	71-80	15	Sudden cardiac death	History of hypertension and cardiac arrythmia, but was stable and asymptomatic on a recent follow-up visit.
**13.**	Intermediate Probability	31-40	12	Sudden cardiac death	History of obesity and gastritis, but otherwise no known comorbidities that may have contributed to death.

**14.**	Intermediate Probability	51-60	15	Sudden cardiac death	No known prior medical history. Documentation in the medical records indicate death due to atherosclerotic heart disease with proximal left coronary artery thrombosis
**15.**	Intermediate Probability	41-50	11	Sudden cardiac death	No known comorbidities that may have contributed to death.
**16.**	Intermediate Probability	41-50	16	Non-ST elevation myocardial infarction	History of polycystic kidney disease presented with chest pain followed by cardiac arrest.
**17.**	Intermediate Probability	31-40	10	Sudden cardiac death	History of hypertension. No other known comorbidities that may have contributed to death.
**18.**	Intermediate Probability	41-50	9	Sudden cardiac death	History of asthma but no other known comorbidities that may have contributed to death.
**19.**	Intermediate Probability	31-40	12	ST-elevation acute anterolateral wall myocardial infarction-	No known comorbidities that may have contributed to death. Presented with typical symptoms of acute coronary syndrome.
**20.**	Intermediate Probability	31-40	6	ST-elevation acute anterior wall myocardial infarction	History of diabetes mellitus. Suffered out-of-hospital cardiac arrest with preceding symptoms of acute coronary syndrome.
**21.**	Intermediate Probability	51-60	8	ST-elevation acute inferior wall myocardial infarction	No known comorbidities that may have contributed to death.
**22.**	Intermediate Probability	51-60	9	Sudden cardiac death	No known comorbidities that may have contributed to death.
**23.**	Intermediate Probability	41-50	14	Sudden cardiac death	No known comorbidities that may have contributed to death. Suffered out-of-hospital cardiac arrest. Subsequent evaluation suggested possible acute ST-elevation myocardial infarction.
**24.**	Low Probability	51-60	6	Sudden cardiac death likely due to atherosclerotic heart disease	Patient with known atherosclerotic heart disease and history of coronary artery bypass grafting
**25.**	Low Probability	51-60	3	Septic shock and multi-organ failure due to *Klebsiella pneumoniae* bacteremia	History of diabetes mellitus, found collapsed at home. Septic shock with *Klebsiella pneumoniae* bacteremia and subsequent multi-organ failure.
**26.**	Low Probability	71-80	5	Sudden cardiac death	History of dementia, chronic pressure ulcers, found collapsed at home.
**27.**	Low Probability	51-60	15	Sepsis due to *Enterobacter cloacae* bacteremia	History of atherosclerotic heart disease, hypertension, ischemic cardiomyopathy with low ejection fraction, presented with decompensated heart failure. Diagnosed with *Enterobacter cloacae* bacteremia, acute kidney injury, and ischemic hepatitis.

**28.**	Low Probability	61-70	20	Sudden cardiac death	Remote history of aortic valve replacement. History of hypertension, well controlled.<br><br>No other comorbidities that may have contributed to death.
**29.**	Low Probability	71-80	18	Sudden cardiac death	History of diabetes mellitus, hypertension, chronic kidney disease.
**30.**	Low Probability	61-70	13	Hypoxic brain injury with associated subarachnoid hemorrhage.	Remote history of aortic valve replacement. History of hypertension and diabetes mellitus;, well controlled.<br><br>Suffered out-of-hospital cardiac arrest of unknown cause.
**31.**	Low Probability	61-70	11	Sudden cardiac death	History of hypertension, diabetes mellitus, chronic kidney disease requiring maintenance hemodialysis. Suffered out-of-hospital cardiac arrest most likely secondary to numerous underlying comorbidities.
**32.**	Low Probability	71-80	19	Severe COVID-19 Pneumonia	History of hypertension, diabetes mellitus, dyslipidemia, hypothyroidism. Developed COVID pneumonia.
**33.**	Low Probability	61-70	19	Pulmonary edema due to decompensated heart failure	Known case of atherosclerotic heart disease, coronary artery bypass grafting, with decompensation of heart failure.
**34.**	Low Probability	61-70	24	Sudden cardiac death	History of hypertension, diabetes mellitus, and heart failure with reduced ejection fraction.
**35.**	Low Probability	41-50	16	Sudden cardiac death	History of hypertension, diabetes mellitus, dyslipidemia, tobacco use.
**36.**	Low Probability	31-40	13	Sudden cardiac death	History of diabetes mellitus and atherosclerotic heart disease, poor adherent to treatment. Suffered out-of-hospital cardiac arrest most likely secondary to numerous underlying comorbidities.
**37.**	Low Probability	31-40	23	Sudden cardiac death	History of uncontrolled diabetes with vascular and neuropathic complications, poor adherence.
**38.**	Low Probability	31-40	30	Sudden cardiac death	No known comorbidities that may have contributed to death. Suffered out-of-hospital cardiac arrest of unknown cause.
**39.**	Low Probability	41-50	16	Hospital associated pneumonia and sepsis due to *Klebsiella pneumoniae* urinary tract infection	History of hypertension, diabetes mellitus and atherosclerotic heart disease, presented with possible ST-elevation acute myocardial infarction. Developed hospital associated pneumonia and urinary tract infection due to *Klebsiella pneumoniae.*
**40.**	Low Probability	41-50	19	Sudden cardiac death	No prior medical records available to ascertain contributory cause(s) of death.

**41.**	Low Probability	51-60	18	Sudden cardiac death	No known comorbidities that may have contributed to death. History of chest pain prior to presentation suggestive of acute coronary syndrome
**42.**	Low Probability	41-50	28	Sudden cardiac death	No known comorbidities that may have contributed to death. Suffered out-of-hospital cardiac arrest of unknown cause.
**43.**	Low Probability	61-70	17	Sudden cardiac death	History of hypertension, diabetes mellitus, chronic kidney disease, dyslipidemia, asthma, obesity, found unresponsive at home.
**44.**	Low Probability	61-70	18	Severe community acquired pneumonia with pleural effusion	History of hypertension, diabetes mellitus and dilated cardiomyopathy with low ejection fraction, sarcoidosis, admitted with community acquired pneumonia with pleural effusion.
**45.**	Low Probability	41-50	21	Sudden cardiac death	No known comorbidities that may have contributed to death. Suffered out-of-hospital cardiac arrest of unknown cause.
**46.**	Low Probability	31-40	19	Sudden cardiac death possibly due to acute myocardial infarction	No known comorbidities that may have contributed to death. Suffered out-of-hospital cardiac arrest with symptoms suggestive of acute coronary syndrome.
**47.**	Low Probability	41-50	20	Sudden cardiac death	No prior medical records available to determine contributory cause(s) of death.
**48.**	Low Probability	21-30	27	Sudden cardiac death	No prior medical records available to determine contributory cause(s) of death.
**49.**	Low Probability	51-60	10	Sudden cardiac death	History of diabetes mellitus and heart failure with reduced ejection fraction, possible atherosclerotic heart disease.
**50.**	Low Probability	31-40	21	Sudden cardiac death likely myocardial infarction	History of hypertension, suffered out-of-hospital cardiac arrest with preceding symptoms suggestive of acute coronary syndrome.
**51.**	Low Probability	51-60	24	ST-elevation anterolateral acute myocardial infarction	No known comorbidities, suffered out-of-hospital cardiac arrest with electrocardiographic changes suggestive of acute coronary syndrome.
**52.**	Low Probability	21-30	22	Sudden cardiac death due to ventricular fibrillation	No known comorbidities, suffered out-of-hospital cardiac arrest with ventricular fibrillation noted by emergency responders.

Supplementary table 2. Number of deaths in Qatar by age and sex between 2019-2021.

Age decile	Female	Male	Not known	Total
0-10*	520	685	112	1317
11-20	30	133	0	163
21-30	59	409	1	469
31-40	133	637	3	773
41-50	165	835	0	1000
51-60	256	829	1	1086
61-70	402	718	0	1120
71-80	466	550	2	1018
81-90	309	400	1	710
91-100	82	88	0	170
100+	13	23	0	36
**Total**	**2435**	**5307**	**120**	**7862**

* This groups includes fetal deaths and miscarriages.
Source: Mortality Review Team, Hamad Medical Corporation, Doha, Qatar.
