# Supplementary material for: Evaluation of mortality attributable to SARS-CoV-2 vaccine administration using national level data from Qatar
Source: Nat Commun. 2023 Jan 3;14:24. doi: 10.1038/s41467-022-35653-z (PMC9808756; doi:10.1038/s41467-022-35653-z)
Supplement: Supplementary file 2 — Reporting Summary [file 41467_2022_35653_MOESM2_ESM.pdf]

nature portfolio

Corresponding author(s): Adeel A. Butt
Last updated by author(s): Dec 8, 2022

Reporting Summary

Nature Portfolio wishes to improve the reproducibility of the work that we publish. This form provides structure for consistency and transparency in reporting. For further information on Nature Portfolio policies, see our Editorial Policies and the Editorial Policy Checklist.

Statistics

For all statistical analyses, confirm that the following items are present in the figure legend, table legend, main text, or Methods section.

n/a	Confirmed	
☐	☒	The exact sample size (*n*) for each experimental group/condition, given as a discrete number and unit of measurement
☐	☒	A statement on whether measurements were taken from distinct samples or whether the same sample was measured repeatedly
☒	☐	The statistical test(s) used AND whether they are one- or two-sided<br>*Only common tests should be described solely by name; describe more complex techniques in the Methods section.*
☐	☒	A description of all covariates tested
☒	☐	A description of any assumptions or corrections, such as tests of normality and adjustment for multiple comparisons
☐	☒	A full description of the statistical parameters including central tendency (e.g. means) or other basic estimates (e.g. regression coefficient) AND variation (e.g. standard deviation) or associated estimates of uncertainty (e.g. confidence intervals)
☒	☐	For null hypothesis testing, the test statistic (e.g. *F*, *t*, *r*) with confidence intervals, effect sizes, degrees of freedom and *P* value noted<br>*Give P values as exact values whenever suitable.*
☒	☐	For Bayesian analysis, information on the choice of priors and Markov chain Monte Carlo settings
☒	☐	For hierarchical and complex designs, identification of the appropriate level for tests and full reporting of outcomes
☒	☐	Estimates of effect sizes (e.g. Cohen's *d*, Pearson's *r*), indicating how they were calculated

Our web collection on statistics for biologists contains articles on many of the points above.

Software and code

Policy information about availability of computer code

Data collection	Data were collected and stored in Microsoft Excel for Microsoft 365.
Data analysis	Data were analyzed in Microsoft Excel for Microsoft 365.

For manuscripts utilizing custom algorithms or software that are central to the research but not yet described in published literature, software must be made available to editors and reviewers. We strongly encourage code deposition in a community repository (e.g. GitHub). See the Nature Portfolio guidelines for submitting code & software for further information.

Data

Policy information about availability of data

All manuscripts must include a data availability statement. This statement should provide the following information, where applicable:

- Accession codes, unique identifiers, or web links for publicly available datasets
- A description of any restrictions on data availability
- For clinical datasets or third party data, please ensure that the statement adheres to our policy

The national dataset used for this study is the property of the Qatar Ministry of Public Health that was provided to the researchers through a restricted-access agreement that prevents sharing the dataset with a third party or publicly. Future access to this dataset can be considered through a direct application for data access to Her Excellency the Minister of Public Health (https://www.moph.gov.qa/english/Pages/default.aspx).

Human research participants

Policy information about studies involving human research participants and Sex and Gender in Research.

Reporting on sex and gender	Data on sex have been reported in the manuscript.
Population characteristics	A total of 6,928,359 SARS-CoV-2 vaccine doses had been administered in Qatar. Over 99% of the vaccines administered were the mRNA vaccines (BNT-162b2 and mRNA-1273). A total of 4,413 deaths were recorded during the study period, with 1,726 deaths occurring any time after at least one dose of a SARS-CoV-2 vaccine (162 deaths after1 dose, 1,354 deaths after 2 doses, and 210 deaths after 3 doses).
Recruitment	Participants were identified from the Qatar National COVID-19 database.
Ethics oversight	The study was approved by the Institutional Review Board at Hamad Medical Corporation. A waiver of informed consent was granted for the study since only decedents records were reviewed and all identifiers were subsequently removed.

Note that full information on the approval of the study protocol must also be provided in the manuscript.

Field-specific reporting

Please select the one below that is the best fit for your research. If you are not sure, read the appropriate sections before making your selection.

[x] Life sciences [ ] Behavioural & social sciences [ ] Ecological, evolutionary & environmental sciences

For a reference copy of the document with all sections, see nature.com/documents/nr-reporting-summary-flat.pdf

Life sciences study design

All studies must disclose on these points even when the disclosure is negative.

Sample size	All decedents during the study period who received any SARS-CoV-2 vaccine were reviewed.
Data exclusions	Those who died more than 30 days after vaccine administration were excluded.
Replication	Four reviewers independently reviewed each medical record to determine and assign the cause of death.
Randomization	N/A
Blinding	N/A

Reporting for specific materials, systems and methods

We require information from authors about some types of materials, experimental systems and methods used in many studies. Here, indicate whether each material, system or method listed is relevant to your study. If you are not sure if a list item applies to your research, read the appropriate section before selecting a response.

Materials & experimental systems

n/a	Involved in the study	
[x]	[ ]	Antibodies
[x]	[ ]	Eukaryotic cell lines
[x]	[ ]	Palaeontology and archaeology
[x]	[ ]	Animals and other organisms
[ ]	[x]	Clinical data
[x]	[ ]	Dual use research of concern

Methods

n/a	Involved in the study	
[x]	[ ]	ChIP-seq
[x]	[ ]	Flow cytometry
[x]	[ ]	MRI-based neuroimaging

Clinical data

Policy information about clinical studies

All manuscripts should comply with the ICMJEguidelines for publication of clinical research and a completedCONSORT checklist must be included with all submissions.

Clinical trial registration	N/A. This is not a clinical trial.
Study protocol	N/A
Data collection	The study was conducted in the State of Qatar between January 1, 2021 and June 12, 2022.
Outcomes	Among the 138 deaths evaluated, 8 deaths were classified as having high probability of being related to vaccination (1.15 death/1,000,000 vaccine doses administered). Fifteen deaths were classified as having intermediate probability (2.16 death/1,000,000 vaccine doses administered). One hundred and twelve deaths were classified as low probability or not related to vaccination.
